# The evolution of preclinical models for myelodysplastic neoplasms

**DOI:** 10.1038/s41375-024-02181-2

**Published:** 2024-02-23

**Authors:** Alain Mina, Steven Pavletic, Peter D. Aplan

**Affiliations:** 1 Myeloid Malignancies Program, Immune Deficiency Cellular Therapy Program, Center for Cancer Research, National Cancer Institute, National Institutes of Health, Bethesda, MD USA; 2 Genetics Branch, Center for Cancer Research, National Cancer Institute, National Institutes of Health, Bethesda, MD USA

**Keywords:** Myelodysplastic syndrome, Cancer models

## Abstract

Myelodysplastic Neoplasms (MDS) are a group of clonal disorders characterized by ineffective hematopoiesis and morphologic dysplasia. Clinical manifestations of MDS vary widely and are dictated in large part by a range of genetic aberrations. The lack of robust in vitro models for MDS has limited the ability to conduct high throughput drug screens, which in turn has hampered the development of novel therapies for MDS. There are very few well-characterized MDS cell lines, and the available cell lines expand poorly in vitro. Conventional xenograft mouse models can provide an in vivo vessel to provide growth of cancer cells, but human MDS cells engraft poorly. Three-dimensional (3D) scaffold models that form human “ossicles” represent a promising new approach and can reproduce the intricate communication between hematopoietic stem and progenitor cells and their environment. Genetically engineered mice utilize specific mutations and may not represent the entire array of human MDS; however, genetically engineered mice provided in vivo proof of principle for novel agents such as luspatercept, demonstrating the clinical utility of this approach. This review offers an overview of available preclinical MDS models and potential approaches to accelerate accurate clinical translation.

## Introduction

Myelodysplastic neoplasms (MDS), also referred to as myelodysplastic syndromes and abbreviated MDS, are a group of malignant disorders of hematopoietic differentiation that are characterized by ineffective hematopoiesis and morphologic dysplasia, leading to abnormal peripheral blood counts, infectious complications, and increased risk of transformation to acute myeloid leukemia (AML) [1–3]. MDS represents a complex disease entity whose pathogenesis stems from an interplay between microenvironment, genetic and epigenetic elements. Compared to AML, advances in MDS therapies are lagging, in large part due to the paucity of successful preclinical models capable of reproducing the complex genetic landscape of this disease. MDS hematopoietic stem cells (HSCs) do not show long term proliferation in vitro [4] and available MDS cell lines are scarce [4]. In addition, xenotransplantation of human MDS cells in immunocompromised murine hosts has been limited by poor engraftment and maintenance of MDS in the host [5] not to mention the inability to reproduce a human bone marrow microenvironment (BME). Other efforts that generated genetically engineered mice (GEM) based on recurrent mutations identified in MDS patients failed to fully replicate all features of human MDS with numerous clinical, morphologic, and genetic aspects of the disease often lacking [6, 7] This made developing therapeutic strategies targeting recurrent genetic abnormalities very challenging. More recent efforts attempted to create “humanized” 3D structures that can mimic the human BME [8, 9]. These models can be quite sophisticated but are far from perfect tools as they require high level resources and expertise. In this synopsis, we will review the evolution of preclinical models from the simple single cell in-vitro systems to the complex in-vivo 3D models.

## In vitro MDS cell lines

Numerous investigators have attempted to generate MDS cell lines, however, these efforts have been largely unsuccessful. An ideal MDS cell line would generate consistent and reproducible results, be easily transferable, and interactive with any artificial matrix that models the BME. Drexler et al. reviewed 31 putative MDS cell lines and used genomic DNA profiling and cytogenetic analysis, to assign each cell line into one of three categories: (1) false/non -malignant cell lines, (2) malignant cell lines in the leukemic phase and (3) valid MDS cell lines (3 of 31) [4]. Cell lines considered a valid representation of the MDS phenotype were M-TAT, TER-3, MDS92, and their derivatives (Table 1).

**Table 1:** Candidate MDS cell lines.

In-vitro MDS Cell Lines	Year	Source	Characteristics	References
MDS92	1991	Bone marrow of 52-year-old with MDS-RARS	-Responsive to cytokines: GM-CSF, IL3	Tohyoma et al. [11]
			-Able to be maintained for prolonged periods in-vivo	
			-Complex cytogenetics (5q-, monosomy7, point mutation in N-RAS)	
M-TAT	1994	Peripheral blood of 3-year old patient with RAEB-MDS	-Responsive to cytokines: EPO, GM-CSF, SCF, IL3	Minegishi et al. [10]
			-Can differentiate into erythroid or megakaryocytic lineages	
MDS-L	2000	MDS92	-Responsive to cytokines (mainly IL3)	Matsuoka et al. [13]
			-Complex cytogenetics (includes 5q-)	
			-Contributed to study of lenalidomide	
			Standard cell line for MDS therapeutic development	
TER-3	2002	Patient with RAEB	-Responsive to cytokines: G-CSF, GM-CSF, IL3, TPO, M-CSF, SCF	Mishima et al. [16]
			-Myeloid and lymphoid surface makers	
			-Complex cytogenetics (monosomy 7, monosomy 20)	
			-Can differentiate into erythroid and megakaryocytic lineages	
MDS-L-2007	2018	MDS92	-Only responds to high dose (100 ng/mL) IL3	Kida et al. [12]
			-H3-K27M mutated	
			-IL-3 dependency depends on H3-K27M status	
MDS-LGF	2018	MDS92	-Responds to low dose (1 ng/mL) IL3	Kida et al. [12]
			-Successfully transplanted in nsgs xenograft models	
			-Provided an early in-vivo mouse model	
			-H3-K27M wild-type	
			-IL-3 dependency depends on H3-K27M status	

The M-TAT cell line was isolated from the peripheral blood of a 3-year-old patient with refractory anemia with excess blasts MDS (RAEB-MDS). These cells were responsive to various cytokines (such as erythropoietin (EPO) and granulocyte-macrophage colony-stimulating factor (GM-CSF)) for growth and maturation of their megakaryocytic and erythroid lineages [10].

The MDS92 cell line was derived from the bone marrow of a 52-year-old patient with refractory anemia and ringed sideroblasts (MDS-RARS) [11]. These cells were not in the leukemic stage and were able to be maintained in vivo for prolonged periods of time while retaining the ability to grow and mature with characteristics that are consistent with MDS. In addition, MDS92 cells proliferated in response to interleukin-3 (IL3) and were characterized by the deletion of 5q chromosome [del(5q)], monosomy 7 and a point mutation in codon 12 of the N-RAS oncogene [11, 12]. A blastic line, MDS-L, was later derived from MDS92 and was instrumental for the study of MDS with del(5q) as well as delineation of the therapeutic effects of lenalidomide on del(5q) MDS [13–15]. Additional blastic sublines were independently isolated from MDS92 and include the MDS-L-2007 and MDS-LGF. Kida et al. described these two major subclones of MDS-L and demonstrated that MDS-L-2007 proliferated in response to high dose (100 ng/mL) IL-3 while the MDS-LGF proliferated in response to much lower doses (1ng/mL). Authors also provided data on the relationship between histone H3 K27M mutation and IL-3 dependence. This data provided crucial evidence that the survival of MDS clones depends on an interplay between environmental factors and intrinsic genetic and epigenetic properties [12].

In 2002, Mishima et al. described TER-3, a new hematopoietic cell line derived from a patient that had recently progressed from refractory anemia (RA) to RAEB [16]. The TER-3 cell line had a complex karyotype and, similar to MDS92, displayed monosomy 7 and no chromosome 5 rearrangements. Cells were found to be constitutively dependent on cytokine signaling for growth and had the potential to differentiate into erythroid and megakaryocytic lineages [16]. Ultimately, Drexler and colleagues concluded that, given its availability and cytogenetic profile that includes a 5q deletion, the MDS92 cell line seemed to be the most promising in vitro MDS model [4].

Given the limited proliferative potential of MDS cell lines as well as the scarcity of dependable in vitro models, the study of genetically engineered murine models and xenografted mice emerged as alternate approaches. These in vivo models may provide a more realistic representation of human MDS as they allow the interaction between mammalian cells and their microenvironment.

## Xenograft models

Patient-derived xenografts (PDX) represent an additional option to reproduce disease complexity in vivo and test therapeutic interventions. To generate a successful PDX model, an immunodeficient recipient is required to avoid immune rejection of the human cells. A landmark study demonstrated that a small subset of AML cells could be transplanted to *Scid* (severe combined immune deficient) mice; this study introduced the concept of cancer stem cells [17]. Combination of a non-obese diabetic (NOD) and severe combined immunodeficiency (SCID) strains resulted in mice that had defective T, B, and NK function [16, 17]. These models were further enhanced by removal of the common gamma chain of the interleukin 2 receptor (IL2rδ^null^) resulting in NOD SCID Gamma (NSG) mice that were more permissive recipients and allowed for significantly higher rates of AML engraftment [18–20].

Despite improved engraftment of AML cells in NSG mice, engraftment of MDS cells remained poor, therefore, additional alterations were made to improve the BME and support trilineage maturation of human progenitor cells [21, 22]. Introducing human cytokine genes into the NSG mouse genome led to the constitutive expression of stem cell factor (SCF), IL3 and granulocyte monocyte colony-stimulating-factor (GM-CSF) resulting in engraftment and expansion of myeloid cells (NSGS mice) [23, 24]. However, most MDS subtypes (except chronic myelomonocytic leukemia) did not engraft [25, 26]. A similar approach was used to generate MISTRG mice, in which human alleles of, macrophage-CSF (M-CSF), IL3, GM-CSF, signal regulatory protein alpha (SIRPα) and thrombopoietin (TPO) replaced their murine counterparts via homologous recombination; these alleles were then crossed onto an immunodeficient background (Rag^−/−^IL2rg^−/−^). This resulted in robust but transient engraftment of CD33+ myeloid cells and some cases of MDS [27–30]. The MISTRG model is promising in that engraftment was not limited to any specific MDS subtype, and MISTRG MDS PDX mice reliably reproduced patients’ dysplastic features and captured their mutational profiles and genetic complexities.

Potential pre-clinical utility of PDX models has been demonstrated by several investigators. In one study, MISTRG mice were engrafted with human MDS cells expressing an IDH2 R140Q mutant protein and treated with an oral IDH2 inhibitor. This resulted in differentiation of IDH2 mutant blasts and myeloid differentiation of the engrafted MDS cells [30]. Although there was no report of survival benefit or improved peripheral blood counts in the treated mice, these observations demonstrate the potential for this model in evaluating innovative and targeted therapies, alone or in combination. An additional study focused on treating mice engrafted with high risk MDS cells using omacetaxine mepesuccinate, a protein synthesis inhibitor [31]. These investigators showed a decrease in engraftment following omacetaxine treatment, which could be potentiated by adding 5-azacytidine or venetoclax. As an alternate to small molecules, antibodies to CD117 [the receptor for SCF] was shown to deplete human MDS stem cells using an in vivo xenograft model [32].

### “Humanizing” the BM microenvironment

**Fig. 1:**
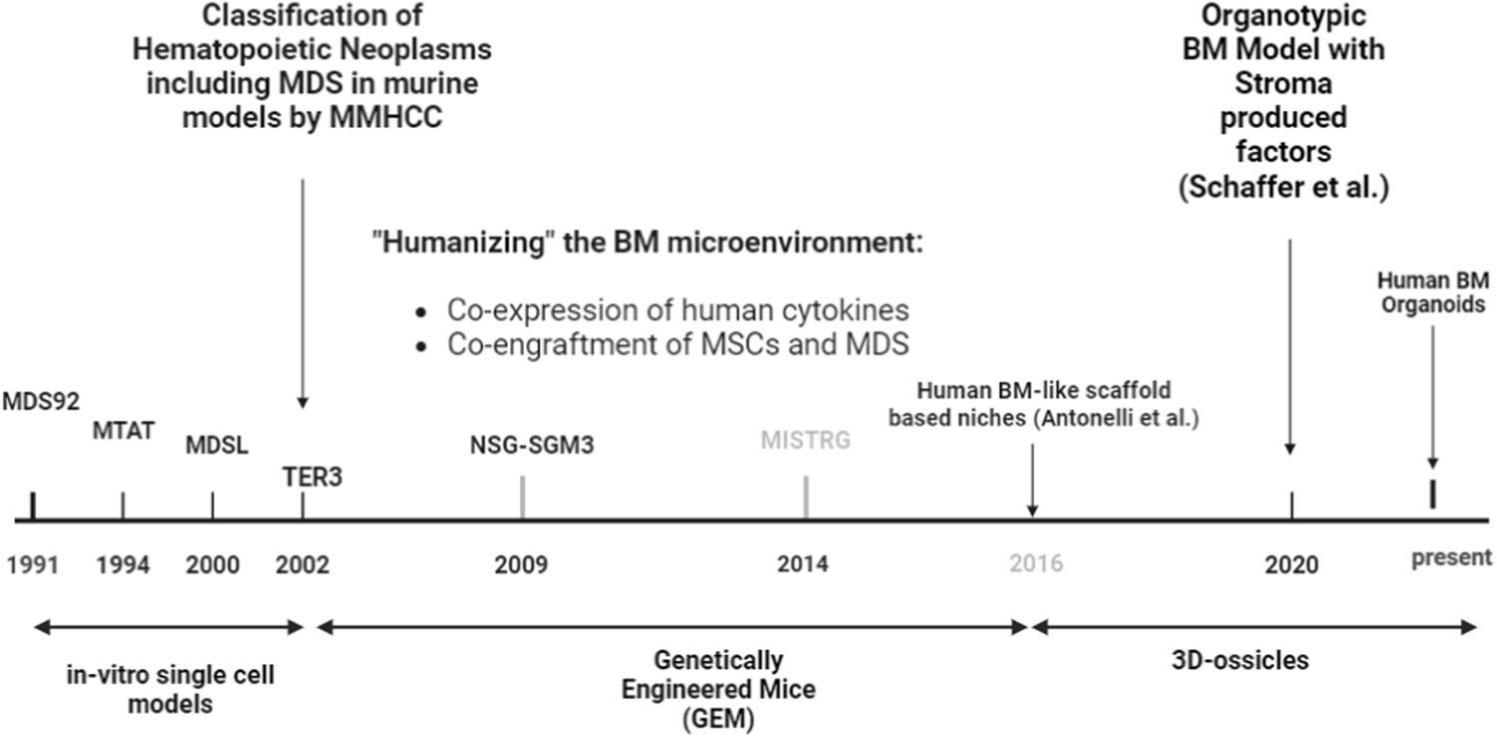
Evolution of preclinical models for MDS timeline. Created with BioRender.com.

Generation of a reliable and readily transplantable MDS PDX remains a challenge due to inadequate engraftment and poor maintenance of donor MDS progenitor cells in the murine host. Some studies suggest a role for the BME in supporting MDS stem cell proliferation and differentiation [33]. This permissive “milieu” relies on the presence of human cytokines and mesenchymal stromal cells (MSCs), both of which have been shown to help engraft MDS cells under specific conditions [33–36]; a summary of humanized approaches is displayed in Fig. 1. Several research groups have attempted to co-engraft MSCs and MDS cells to promote stem cell myeloid differentiation and persistence. In one study, CD34+ bone marrow cells were intrafemorally injected alongside human stromal cells into NOG (NOD/Shi-*scid*/IL-2Rγ^null^) mice and the percentage of human CD45+ engraftment was as high as 89% with suppression of murine host hematopoiesis [35]. Similarly, Medyouf et al. demonstrated that the co-injection of patient-derived MDS CD34+ cells with MSCs led to efficient engraftments (70%) in NSG mice with the resulting xenograft model displaying dysplasia and characteristic molecular lesions [36]. This approach has been used as a pre-clinical platform to assess the efficacy of eltrombopag in producing platelet support for MDS in vivo [37], as well as to assess the ability of PXS-5505 (a pan-lysyl oxidase inhibitor) and 5-azacytidine to augment erythroid differentiation in vivo [38].

A common denominator of these models was sub-optimal, but improved, engraftment, leading to the concept that a BME that more closely mimicked human bone marrow might improve engraftment of primary MDS cells [39, 40]. The reasons behind sub-optimal engraftment are many and likely related to species-specific environmental requirements that promote stem cell survival, homing, and engraftment [23].

HSCs reside and flourish in the bone marrow and interact with an intricate network of cells that include MSCs, Schwann cells, vascular endothelial cells, and osteoblasts, all of which are necessary for proper function of HSCs [33, 41]. These “niches” are thought to support both hematopoietic and leukemic stem cells. Transfer of a scaffold structure that combines human hematopoietic BM cells with nonhematopoietic cells (referred to as an “ossicle”) into an immunodeficient mouse, can generate a “humanized” BM niche [8, 9, 42]. Reinisch et al. developed a humanized BMME by subcutaneous injection of extracellular matrix material with immature MSCs leading to ossicle formation in vivo. Following daily injection of human parathyroid hormone, these ossicles were able to support engraftment of human CD34+ hematopoietic stem and progenitor cells (HSPC). Compared to unmanipulated NSG mice, these “humanized” ossicles led to more robust engraftment of HSPCs as well as AML, acute promyelocytic leukemia (APL), and myelofibrosis samples [8].

Altrock et al. evaluated the feasibility of this approach [8, 9] in MDS and used a standardized approach to compare the 3D ossicle-based method to intrafemoral (IF) co-injection of MSCs and CD34+ HSPCs [41]. A combination of immunohistochemistry, flow cytometry and molecular analysis demonstrated that 3D humanized ossicle xenotransplantation had a significantly better engraftment rate of MDS samples compared to the historical IF injection of MSCs [36, 43]. A potential drawback of the 3D model was the poor recovery rates of MDS cells from the humanized matrices, which would impede downstream functional and molecular analyses. They suggested immortalizing matrix components and adding endothelial cells to help alleviate that concern [40, 41].

Additional studies have used the concept of 3D scaffold generation to produce ceramic matrices coated with MDS MSCs which were subsequently implanted into immunodeficient mice. The resulting human niche was associated with engraftment of AML samples across all major molecular and risk subgroups [44]. Abarrategi et al. used a gelatin-based scaffold to test distinct implantable niches (osteoblastic, endothelial and MSCs). The authors found that use of this scaffold was associated with improved engraftment of human AML samples compared with other approaches; it is important to note that these studies used human AML and not MDS samples [45]. Another study used a gelatin-based scaffold to create an in vivo vascularized disease model that allowed engraftment of 94% of MDS HSC patient samples irrespective of MDS subtype [46]. Of note, disease-associated “aging” was also demonstrated in this model with a trend toward myeloid differentiation, particularly, in high-risk samples [46].

In addition to vascularizing the niche, co-culturing of HSPCs with MSCs, and adding a 3D structural matrix, other elements of the human BM have been used to help mimic the dynamic interaction between hematopoietic cells and microenvironment. Khan et al. developed vascularized “living” organoids that incorporated human pluripotent stem cells that could generate myeloid and mesenchymal elements as well as vascular sinusoidal structures that can mimic the 3D architecture of the bone marrow [47]. Multimodal imaging and single cell RNA sequencing were used to demonstrate similarity between these organoids and human BMME. These organoids were also shown to support engraftment and proliferation of healthy and malignant human cells [47]. Proof of disease reproducibility was provided when organoid “remodeling and fibrosis” occurred after engraftment of myelofibrosis patients’ cells though application of fibrosis inhibitors such as TGFβ and BET inhibitors and ruxolitinib did not reverse hallmarks of fibrosis. Although MDS was not evaluated with this model, other diseases included were AML, acute lymphoblastic leukemia (ALL), chronic myelogenous leukemia (CML) and multiple myeloma [47]. Despite an impressive homology between this organoid’s vascular system and the human BM endothelial system, this model failed to reproduce adipocytes, lymphocytes and smooth muscle cells, limitations that likely could be enhanced by optimizing the growth factors, chemokines, and cytokines in this “hematopoietic milieu”.

## Genetically engineered mice

In 1999, The Mouse Models of Human Cancers Consortium (MMHCC) emerged as an effort by the National Cancer Institute to develop mouse models of cancer that were corroborated by the scientific committee and used to better understand different pathways and processes involved in cancer development [48]. In 2002, the hematopathology subcommittee of the MMHCC, developed a consensus recommendation for the classification of hematopoietic neoplasms, including MDS, in murine models. Using clinical course, blood counts, histopathology and immunophenotypic features, they identified myeloid dysplasia which was defined by the presence of cytopenias in the peripheral blood of mice as well as dysplasia in at least 1 of the 3 myeloid lineages [48, 49]. Unlike human MDS, where the presence of dysplasia in 10% of a particular lineage, continues to hold as a diagnostic threshold, the minimal required dysplasia in mice is yet to be defined or validated. Nonetheless, this classification has provided scientists, pathologists, and investigators, with a standardized tool to diagnose MDS in GEM allowing these models to be reliably used to test novel diagnostic and treatment approaches to cancer management.

There are several approaches that have been used to generate MDS in mice; these approaches are distinct from xenografts, which engraft human MDS cells into mouse hosts. The first includes treating mice with known mutagens, such as benzene [50, 51], alkylating agents [52, 53] or viruses [54]. These strategies resulted in a wide spectrum of complex genetic phenotypes including MDS. However, despite providing insight into certain phenotypic aspects of MDS, these mutagenic approaches were largely unable to reliably define contributions of particular genes. In addition, since these approaches depended largely on random mutagenic events, they were not readily reproducible.

Two additional approaches are based on using mutations identified in patients with MDS to generate genetically engineered HSPC. Both approaches have been successful in replicating some, but not all, features of MDS, with certain clinical, morphological, or genetic aspects of the murine model disease lacking when compared to human MDS. In the first approach, murine HSPC are modified to express a mutant gene in vitro, typically using retroviral or lentiviral vectors. The modified HSPC are then transplanted into syngeneic hosts that have been lethally irradiated. Given the requirement for transplantation, this approach is primarily limited to the study of hematologic malignancy, and not easily adapted to the study of solid tumors, such as lung cancer.

A second general approach includes the creation of GEM models that have modified the germline mouse DNA. Transgenic, “knock-out” or “knock-in” mice, can be produced by a variety of techniques, including pronuclear injection of produced by injecting DNA into a fertilized egg or by homologous recombination of embryonic cells followed by blastocyst injection [55]. The general technique for generating GEM models has been refined and modified over the past forty years to allow use of endogenous promoters, as well as tissue specific and temporal expression of mutant genes; advantages and disadvantages for several of these modifications are listed in Table 2. These manipulations lead to modification of germline DNA, which can then be transmitted from generation to generation. A large cohort of mice with an identical genetic modification can then be observed to determine the incidence of MDS in the modified mice.

**Table 2:** Pros and cons of genetically engineered mice (GEM).

Technique	Pros	Cons
Transgenic using ubiquitous promoter	Initial technique used to generate GEM	-Random integration effect
		-Ubiquitous, non-physiologic expression
Transgenic using tissue specific promoter	Tissue specific expression	-Random integration effects
		-Expression may be non-physiologic
Homologous recombination “knock-out”	Useful for gene inactivation	-Vector, targeting can be challenging, unpredictable.
		-Gene inactivated in all tissues.
		-CRISPR improvement.
Homologous recombination “knock-in”	Express mutant cDNA, fusion gene	-Same as knock-out.
	Physiologic expression from endogenous promoter.	-Expression of mutant in non-relevant tissue (e.g. ldh2 KI)
Conditional knock-out/knock-in plus	Target knockout to specific tissue	-More complex breeding.
Tissue specific Cre (trigger for event)		-Tissue specific expression may be "leaky"
Conditional knock-out/knock-in plus	Trigger Cre expression post-natal	-More complex breeding
time specific Cre (eg, Mx1, CreERT2)		-Time specific expression may be leaky

Although there are several advantages of using GEM models, such as knowledge of specific genes modified, transferability of GEM between laboratories, reproducibility, and ability to generate large numbers of genetically identical mice, there are several disadvantages as well. The mouse hematopoietic system, while similar to human, is not identical [56]. These mice are generally kept in specific pathogen free environments, which may not adequately mimic the diverse microbiome seen in humans. In addition, many investigators prefer to use inbred mice, which simplifies genetic analysis and facilitates HSC transplant experiments. This strategy may not adequately recapitulate human genetic diversity. Finally, single engineered mutations may be insufficient to adequately mimic the heterogeneity seen in human MDS, which may require combinations of mutant genes to produce a phenotype. In fact, several highly penetrant GEM models of leukemia are linked to spontaneous acquisition of somatic mutations, which collaborate with the mutation engineered in the mouse germline [57, 58].

### TET2

Somatic mutations in the ten-eleven translocation 2 (*TET2*) gene, leading to loss of function, are found in up to 20% of MDS patients [59]. *TET2* was shown to play a role in hematopoietic development, myeloid differentiation [60] and when mutated, malignant transformation [61–63]. Transplantation of *Tet2*^−/−^ cells into lethally irradiated mice resulted in a CMML-like disease characterized by myeloid dysplasia in bone marrow as well as splenomegaly, neutrophilia, monocytosis and extramedullary hematopoiesis. *Tet2* monoallelic loss had a similar but less pronounced phenotype [62].

### EZH2

*EZH2* is a histone methyltransferase located on chromosome 7 and found to be inactivated in clonal myeloid disorders such as MDS and MPN [64, 65]. *Ezh2* knockout mice (*Ezh2*^−/−^) developed myelodysplastic disorders characterized by anemia, splenomegaly, and dysplasia of the bone marrow, and the concurrent loss of both *Tet2* and *Ezh2* resulted in either an MDS phenotype (pancytopenia, myelodysplasia) or MDS/MPN phenotype (monocytosis, splenomegaly) [63]. Interestingly, although latency of disease progression was markedly shortened when both genes were deleted, this genetic combination showed little to no leukemogenic potential. Consistent with observations that *EZH2* mutated MDS have a very low risk of AML progression [66, 67], no *Ezh2*^−/−^ or *Tet2*^−/−^*Ezh2*^−/−^ mice developed AML in their experiments [63].

### U2AF1

Mutations in spliceosome genes are found in more than 80% of MDS patients [68]. Spliceosome gene aberrations are thought to often be an MDS initiating event that arises early in the disease process and disrupts healthy hematopoiesis [69, 70]. Mouse models that express mutant genes based on point mutations that have been identified in patients with MDS have been developed for some of these mutations including *SF3B1, SRSF2* and *U2AF1* [71–74]. Transgenic mice expressing a U2af1 S34F mutation were reported by Shirai et al. [72]. Cells containing a doxycycline-inducible human mutated (mut) *U2AF1* or control *U2AF1* cDNA were transplanted to lethally irradiated recipients. Following engraftment, both mut *U2AF1* and control *U2AF1* recipients received doxycycline [75]. Mice with mutated *U2AF1* treated with doxycycline developed an isolated peripheral leukopenia (normal platelet counts and red blood cells) while their marrows had increased neutrophil count, apoptotic activity and reduced monocytes and B cells. Prolonged follow up after transplantation (500 days) did not reveal any dysplasia, MDS, or AML development [75]. More recently, *U2af1* knock-in models with Cre-dependent knock-in alleles of *U2af1*^*S34F*^ were generated and led to dysplasia, macrocytic anemia and leukopenia, but did not demonstrate decreased survival or increased leukemogenesis [76]. This model helped identify cooperating mutations via a study in which a combination of *U2af1*^*S34F*^ and *Runx1* deficiency led to abnormal hematopoiesis, but not MDS or AML. However, after mutagenesis with ethyl-nitrosourea, a minority of mice developed AML accompanied by mutations in *Ikzf1, Idh1* and *Gata2* [76].

### SF3B1

*SF3B1* mutations characterize a subset of MDS with RA, ring sideroblasts, and favorable outcome [72]. Several animal models have attempted to reproduce certain aspects of *SF3B1* mutated MDS with varying degrees of success. Obeng et al. created an *Sf3b1*^*K700E*^ model in which mice that expressed a *SF3B1*^*K700E*^ “knock-in” allele developed erythroid dysplasia and macrocytic anemia but not clear MDS [77]. Independently, and using a “minigene” approach, Mupo et al. developed an *Sf3b1*^*K700E*^ model that seemed to have an adequate heterozygous allele expression (50% by RNA-seq analysis) but failed to phenotypically produce disease: There was no difference in overall survival between *Sf3b1*^*K700E*^ mice and wild-type, and no mouse developed MDS. Despite progressive normocytic anemia, platelet counts and white blood cell counts remained normal and morphological assessment of the bone marrow revealed no dysplasia or ring sideroblasts [78].

### SRSF2

Mutations in *SRSF2* are found in up to 15% of MDS patients and often associated with poorer outcomes and a shorter duration to leukemic transformation [71, 79]. Kon et al. developed a knock-in model in which Srsf2^P95H/+^ mice developed macrocytic anemia by week 15, along with leukopenia and a marked reduction in progenitor cell population. Dysplastic changes in erythroid and megakaryocytic lineages in the bone marrow were detected, although none of the mice developed MDS or AML by week 90 [80]. Another knock-in model, *Srsf2*^*P95H/+*^ mice, was developed by Smeets et al. who confirmed heterozygous expression and transcription by RNA-seq. *Srsf2*^*P95H/+*^ mice developed findings consistent with MDS by 12 months of age, with evidence of morphologic dysplasia in myeloid and erythroid lineages in bone marrow and peripheral blood, without progression into AML. Gene expression analysis found genetic signatures consistent with myeloid differentiation, loss of lymphoid maturation potential and MDS progression [81]. Unlike previous reports, in which *Srsf2*^*P95H/+*^ cells displayed poor engraftment, Smeets et al. were able to overcome this limitation by modifying the bone marrow competitor [81].

### DICER

There is evidence that MDS pathogenesis can be driven, at least in part, by an interplay between somatic mutations and the BME [82]. This interaction is necessary for disease progression and maintenance of the malignant clone. Mimicking some of the dysfunction observed in the BME of MDS patients has been used to reproduce disease and create models to better understand it. Dicer1 is an RNase III endonuclease that acts to process pre-miRNA and synthesize microRNA necessary to regulate diverse cellular functions including hematopoiesis [83, 84]. *Dicer1* deletion led to global downregulation of microRNAs and drove tumorigenesis in a mouse model of lung cancer mediated by expression of a mutant form of *Kras* [85]. In a similar effort, Raaijmakers et al. generated mice that deleted *Dicer1* in a subset of mesenchymal osteolineage bone marrow cells (designated *Oxs-GFP-Cre*^*+*^*Dicer*^*fl/fl*^ or *OCD*^*fl/fl*^) [86]. *OCD*^*fl/fl*^ mice showed stunted growth with only 70% survival by week 8. Analysis at 4–6 weeks revealed impaired osteoblastic differentiation and decreased bone marrow mineral matrix deposition. Of interest, *OCD*^*fl/fl*^ mice demonstrated ineffective hematopoiesis characterized by decreased peripheral blood counts (marked leukopenia and variable anemia and thrombocytopenia) despite having normal to increased bone marrow cellularity. Morphological examination of their bone marrows revealed dysplastic features, which, along with peripheral cytopenias, were consistent with a diagnosis of MDS per the Bethesda criteria [48, 86]. Other findings that are characteristic of human MDS included increased growth and apoptosis of primitive hematopoietic progenitor cells, decreased B-cell progenitor cells, marked vascularity of the bone marrow and a preferential maturation toward myeloid lineages. In addition, the authors demonstrated the role of BME in MDS pathogenesis by transplanting *OCD*^*fl/fl*^ HSCs from mice with clinical MDS onto healthy wild-type recipients. Despite complete donor chimerism, indicating robust engrafted of the mutant *OCD*^*fl/fl*^ cells, transplant recipients had normal peripheral blood counts and no dysplastic features. Alternatively, when HSCs from healthy wild-type mice were transplanted onto lethally irradiated *OCD*^*fl/fl*^ mice, the wild-type cells developed features of MDS. This series of experiments demonstrated that the *OCD*^*fl/fl*^ mouse model was successful at reproducing clinical MDS and delineating the interplay between disease pathogenesis and BME [86].

### NUP98 translocations and MDS

The *NUP98* gene is present on chromosome 11p15.5 and encodes a component of the nuclear pore complex, which can also function as a transcription scaffold [87]. Fusion proteins resulting from chromosomal translocations involving the *NUP98* gene have been identified in lymphoid and myeloid malignancies and act as oncoproteins that can drive malignant transformation [88, 89]. *NUP98* fused to several clustered homeobox (HOX) genes, including *HOXA9, HOXA13*, and *HOXD13* have been identified in patients with MDS and AML [90]. Pineault et al. generated a *NUP98-HOXD13* murine model in which mouse bone marrow stem cells that expressed a *NUP98-HOXD13* fusion gene were generated via retroviral transduction. Colony forming unit spleen assay analysis of transduced bone marrow cells showed that myeloid cells (Gr+/Mac1+) were increased while erythroid precursors were reduced at day 12. In that study, some *NUP98-HOXD13* mice developed myeloproliferative disease at the 4-week post-transplant mark but none progressed to AML (except those engineered to concurrently express the TALE homeobox gene *Meis1*, a strong mediator of *NUP98*-associated leukemia [91] during a 6-month study period [92].

Lin et al. developed *NUP98-HOXD13 (NHD13)* transgenic mice that utilized the HS21/45-*Vav* vector [93] to direct the expression of a human *NHD13* fusion cDNA in hematopoietic tissues of mice. Like human MDS, *NHD13* mice developed dysplasia and peripheral blood cytopenias in the setting of a hypercellular marrow, demonstrating ineffective hematopoiesis. In addition, this model reproduced the natural progression of MDS, as *NHD13* transgenic mice show mild-moderate anemia and remain healthy for an extended period (typically 6–10 months) but develop progressively worsening cytopenias or transform to acute leukemia, most commonly between 10 and 14 months, with >90% of mice surviving less than 14 months [93]. Progression to acute leukemia was accompanied by spontaneous acquired mutations in genes commonly mutated in human MDS, such as *Nras, Kras, Ptpn11*, and *Cbl* [57].

The MDS seen in *NHD13* mice was cell-autonomous, as WT mice transplanted with *NHD13* bone marrow inevitably developed MDS, characterized by outcompeting WT bone marrow, macrocytic anemia, dysplasia, and transformation to AML. Additional transplant assays analyzed BME cellular elements from *NHD13* mice, and found increased endothelial cells, decreased megakaryocytes and dysfunctional osteoblastic and mesenchymal cell populations [94]. Inflammatory cytokines shown to be increased in human MDS were also elevated in *NHD13* mice suggesting that this model may capture aspects of the interplay between BMME and MDS pathogenesis [94]. In addition, transplantation of MHC matched WT bone marrow into *NHD13* recipients led to increased survival, but not cure, of the transplant recipients, while transplant of MHC mismatched bone marrow led to an increased graft versus tumor effect [95]. The utility of this model for pre-clinical drug development is underscored by the fact that *NHD13* mice were used to provide proof-of-concept for luspatercept, the only new drug to receive FDA approval for MDS in the past decade (100,101).

## Conclusion

Successful MDS models should be reproducible, user-friendly, and dynamic tools that are able to accurately replicate the complex biological features of MDS. Ideal models would be “living” entities that reproduce the intricate communication between HSCs and their environment. In concept, these ideal models would allow one to individualize approach and evaluate treatment regimens while avoiding toxicities. However, although substantial progress has been made, such ideal models do not yet exist. But the MDS research community should not let “the perfect be the enemy of the good.” Despite the lack of ideal models, drugs (such as luspatercept) continue to be developed for patients with MDS using the available, less-than-ideal models described above. With the increased efforts over the past decade comes optimism that increasingly useful and dependable tools which accurately mimic MDS will continue to evolve.

### Disclaimer

The views expressed in this work do not represent the official views of the National Institutes of Health or the United States Government.
